# Platelet Consumption and Filter Clotting Using Two Different Membrane Sizes during Continuous Venovenous Haemodiafiltration in the Intensive Care Unit

**DOI:** 10.1155/2014/203637

**Published:** 2014-04-27

**Authors:** Francesca Bonassin Tempesta, Alain Rudiger, Marco Previsdomini, Marco Maggiorini

**Affiliations:** ^1^Medical Intensive Care Unit, University Hospital Zurich, Raemistrasse 100, 8091 Zurich, Switzerland; ^2^Institute of Anaesthesiology, University Hospital Zurich, Raemistrasse 100, 8091 Zurich, Switzerland; ^3^Intensive Care Unit, Regional Hospital of Bellinzona, 6500 Bellinzona, Switzerland

## Abstract

*Background*. The aim of this study was to investigate whether different haemofilter surface areas affect clotting and platelet consumption in critically ill patients undergoing continuous venovenous haemodiafiltration (CVVHDF). *Methods*. CVVHDF was performed in postdilution technique using a capillary haemofilter with two different membrane sizes, Ultraflux AV 1000S (*n* = 17, surface 1.8 m^2^, volume 130 mL), and the smaller AV 600S (*n* = 16, surface 1.4 m^2^, volume 100 mL), respectively. Anticoagulation was performed with heparin. *Results*. No significant differences were found when the two filters were compared. CVVHDF was performed for 33 (7–128) hours with the filter AV 1000S and 39 (7–97) hours with AV 600S (*P* = 0.68). Two (1–4) filters were utilised in both groups over this observation period (*P* = 0.94). Platelets dropped by 52,000 (0–212,000) in AV 1000S group and by 89,500 (0–258,000) in AV 600S group (*P* = 0.64). Haemoglobin decreased by 1.2 (0–2.8) g/dL in AV 1000S group and by 1.65 (0–3.9) g/dL in AV 600S group (*P* = 0.51), leading to the transfusion of 1 (0–4) unit of blood in 19 patients (10 patients with AV 1000S and 9 with AV 600S). Filter observation was abandoned due to death (12.1%), need for systemic anticoagulation (12.1%), repeated clotting (36.4%), and recovery of renal function (39.4%). *Conclusion*. Our study showed that a larger filter surface area did neither reduce the severity of thrombocytopenia and anaemia, nor decrease the frequency of clotting events.

## 1. Introduction

Acute renal failure (ARF) has a high incidence among critically ill patients, occurring predominantly as a consequence of shock and systemic inflammation, and leads to a prolonged hospital length of stay and considerable costs [1–7]. ARF is related to significant morbidity and is an independent predictor of mortality [8]. Renal replacement therapy (RRT) is the standard of care for this condition and is generally performed as continuous venovenous haemodiafiltration (CVVHDF) in the intensive care unit setting [9]. The correlation between filter characteristics, filter performance, and CVVHDF safety in critically ill patients has rarely been subject of research, despite the long experience of continuous renal replacement therapy (CRRT) in clinical practice [9–14]. CVVHDF related complications, for example, hypotension, electrolytes disturbances, platelet consumption, and hypercoagulable state, potentially lead to an unfavourable outcome among critically ill patients with ARF [9, 15–17]. CVVHDF is also associated with significant risk of bleeding due to the required extracorporal circuit anticoagulation, secondary platelet dysfunction, and thrombocytopenia [9, 15]. Finally, recurrent filter clotting leads to dialysis discontinuation and to frequent filter change, resulting in reduced treatment efficacy and high costs.

In consideration of these aspects, we aimed to review our use of two different haemodiafiltration membrane sizes (1.8 m^2^ versus 1.4 m^2^) and compare the findings with regard to platelet count, haemoglobin level, and filter longevity.

## 2. Methods

### 2.1. Study Population

This retrospective study was performed in the 12-bed medical intensive care unit (ICU) of the University Hospital Zurich, Switzerland, and was approved by the local ethic committee. All consecutive adult patients, who required CVVHDF between November 2007 and June 2009, were screened for the study. Patients with coagulation abnormalities or need for systemic anticoagulation were excluded. Patients were included into the analysis only once, even when they were readmitted during the observation period. Routine clinical information and laboratory parameters, including parameters for haemolysis and coagulation, were collected from the charts. The observation began with the start of CVVHDF and ended when (a) the patient died, (b) the patient did not require CVVHDF anymore, or (c) the standard anticoagulation procedure was abandoned, for example, change to predilution technique or switch to a therapeutic anticoagulation regimen or to anticoagulation with citrate.

Criteria for starting CVVHDF in critically ill patients were preexisting chronic renal failure (on intermittent dialysis), acute renal failure with oliguria (<500 mL/d) and signs of fluid overload according to the RIFLE and AKIN classification, hyperkalaemia (K^+^ > 6.5 mmol/L), metabolic acidosis (pH < 7.20), uremic symptoms, and poisoning with dialysable toxins [1, 6, 18]. Patients with higher bleeding risk or contraindications for heparin anticoagulation, for example, thrombocytopenia, congenital or acquired coagulation disorders, and history of severe bleeding or heparin induced thrombocytopenia (HIT), were primarily assigned to citrate anticoagulation and excluded from our observation.

### 2.2. Instruments and CVVHDF Procedure

CVVHDF was performed with a multifiltrate machine (Fresenius, Homburg, Germany) and two different capillary haemofilters, Ultraflux AV 1000S (*n* = 17) and Ultraflux AV 600S (*n* = 16), were utilised according to their availability. Both filters contained a polysulfone membrane able to eliminate molecules with a weight of up to 30,000 Dalton, AV 1000S having a larger filtrating surface and bigger blood filling volume compared to the smaller AV 600S filter (1.8 m^2^ versus 1.4 m^2^; 130 mL versus 100 mL). According to the manufacturer, wall thickness and inner diameter of the capillaries were the same in both filters. Our standard protocol required an effluent flow rate of 35 mL/kg/h and, in agreement with the accepted practice at the time, the flow rate was increased to 50 mL/kg/h during the first 24 hours in patients with severe sepsis and septic shock [15]. Ultrafiltrate and dialysate (Hemosol B0, Gambro Hospal, Basel, Switzerland) fluids were replaced in a 1 : 1 ratio, and the substitution fluid was given postfilter. The filtration fraction was adjusted to be ≤20% of the blood flow. Anticoagulation was performed with standard unfractionated heparin, given before the filter, the initial dose being 8 U/kg/h. A first analysis of the postfilter activated partial thromboplastin time (aPTT) was performed 2 hours after the start of CVVHDF. The anticoagulation targets were a postfilter and systemic aPTT between 40–50 s and <45 s, respectively. Heparin doses were adjusted accordingly, and aPTT measurement was repeated after 6 hours.

### 2.3. Statistical Analysis

Results are given as median (range) or percentages. Comparisons between the two filter groups were made with the Mann-Whitney *U* test and the chi-square test as appropriate. All testing was two-tailed and a *P* value < 0.05 was considered significant. All statistical analyses were performed with the use of SPSS 17.0 for Mac OS X.

## 3. Results

### 3.1. Patient Characteristics

53 patients were screened and 33 were included in the analysis. Two groups of patients were compared according to the type of filter and there were no significant differences among their baseline characteristics (Table 1).

Main diagnoses on ICU admission were acute heart failure (30.3%), sepsis (27.3%), and acute respiratory failure (12.1%). The remaining 30.3% of patients were admitted due to renal (3.0%), gastrointestinal (9.1%), neurologic (9.1%), and endocrine disease (3.0%) and for postoperative management after elective surgery (6.1%). Half of included patients had a moderate to severely impaired glomerular filtration rate (GFR < 60 mL/min) already before their admission to the ICU (Table 1). All but one had documented ARF or acute-on-chronic deterioration of renal function at CVVHDF start. Sepsis, haemodynamic instability, and toxic renal damage (contrast medium, nonsteroidal anti-inflammatory drug, rhabdomyolysis) were among the precipitant causes in 16 (48.5%), 8 (24.2%), and 5 (15.2%) patients. One patient (3.0%) developed ARF due to progressive minimal change glomerulonephritis and 2 patients (6.1%) as a complication of diabetic and hepatic coma, respectively. One patient (3.0%) only did not show any evidence of renal impairment and required CVVHDF for severe hyperammonemia and related encephalopathy.

CVVHDF was started, while 69.7% of the patients were requiring haemodynamic support with catecholamines and 72.7% were on mechanical ventilation. SAPS II and SOFA scores were 63 (30–98) and 12 (8–16), respectively. ICU and hospital length of stay were 8 (1–168) days and 22 (2–171) days. Overall, ICU mortality reached 33.3% and hospital mortality 42.4% (Table 1).

### 3.2. Filter Comparison and Safety

Baseline laboratory parameters at CVVHDF start were similar between patients treated with AV 1000S and AV 600S filters, with the exception of the potassium level (*P* = 0.03, Table 1). The results of the filter performance analysis are shown in Table 2.

**Figure 1:**
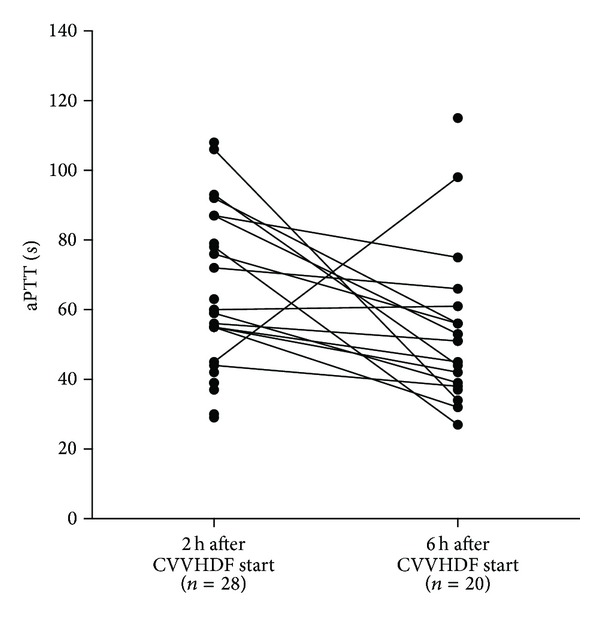
Postfilter aPTT values two and six hours after CVVHDF initiation.

There was no difference in creatinine and blood urea nitrogen (BUN) clearance, or in heparin dose needed for extracorporal circuit patency. During the first six hours of CVVHDF, patients received 450 (0–1050) U/h heparin, representing a dose of 7 (0–9) U/kg/h. Postfilter aPTT 6 hours after starting CVVHDF was 51 (27–115) s. After 6 hours, only 15% of the postfilter aPTT measurements were within our predefined target range of 40–50 s, whereas 30% were below and 55% above this range (Figure 1). Postfilter aPTT measurements were missing in 5 patients after 2 hours and in 13 after 6 hours.

Creatinine and BUN decreased significantly (*P* < 0.001) with CVVHDF, but there was no difference in efficacy between the two filters. Bicarbonate increased by 4.7 mmol/L (23% of the baseline value) to 24 (5.3–29.7) mmol/L at the end of the observation period.

**Figure 2:**
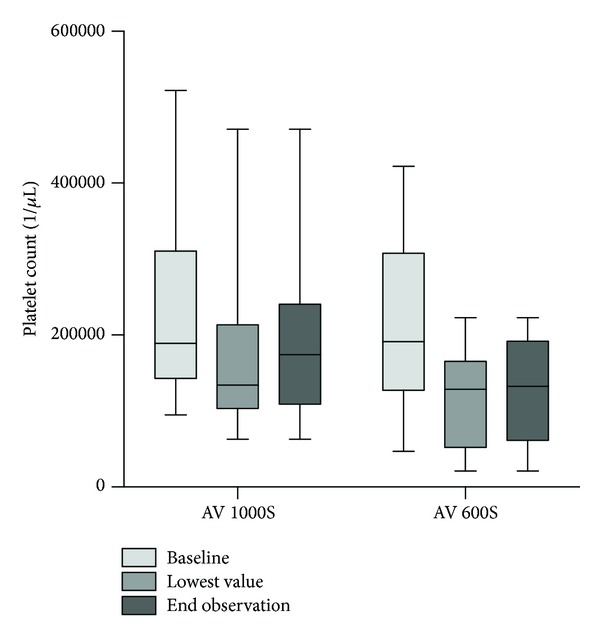
Changes in platelet counts with CVVHDF.

During CVVHDF, thrombocytopenia was a common finding and platelet count dropped by 52,000 (0–212,000) and 89,500 (0–258,000) using AV 1000S and AV 600S filter, respectively (*P* = 0.64, Figure 2).

**Figure 3:**
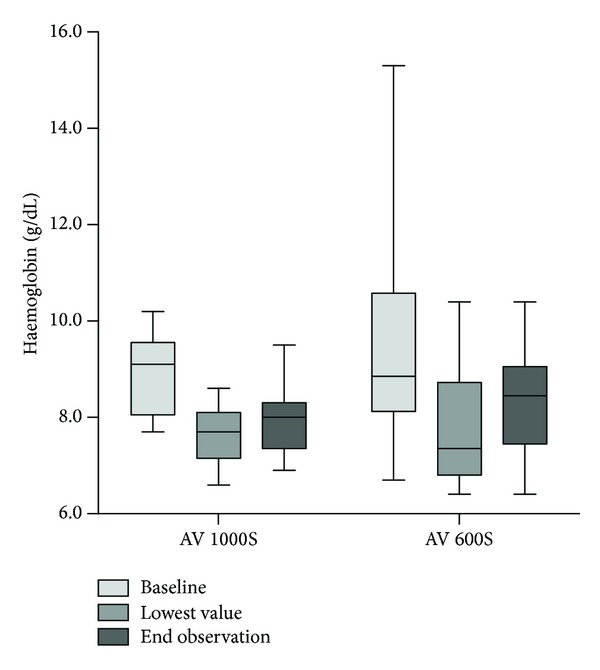
Changes in haemoglobin concentrations with CVVHDF.

Haemoglobin decreased by 1.2 (0–2.8) g/dL in AV 1000S group and by 1.65 (0–3.9) g/dL in AV 600S group (*P* = 0.51, Figure 3), overall to the transfusion of 1 (0–4) unit of red blood cells in 19 patients (57.6%). There were no significant differences between the two filter groups, with 10 patients in AV 1000S and 9 patients in AV 600S group requiring the transfusion of 1 unit of blood (ranges 1–4 and 1–3, respectively, *P* = 0.81). No platelet transfusions were needed and no patient developed a heparin induced thrombocytopenia (HIT).

The overall CVVHDF observation period was 33 (7–128) hours with AV 1000S and 39 (7–97) hours with AV 600S filter (*P* = 0.68), and 2 (1–4) filters were utilised in both groups (*P* = 0.94). Overall filter longevity was 15 (4–67) hours with AV 1000S and 21 (4–42) hours with AV 600S filters (*P* = 0.63). Observation was abandoned due to death (12.1%), need for systemic anticoagulation (12.1%), repeated clotting (36.4%), and recovery of renal function (39.4%). Among the patients requiring continuation of CVVHDF, 33.3% were changed to predilution technique and 19.5% to intermittent renal replacement therapy and 27.7% were started on therapeutic anticoagulation with heparin and 19.5% on citrate anticoagulation.

## 4. Discussion

Our retrospective study did not demonstrate any significant difference related to the filter size but confirmed that CVVHDF is a very effective therapy in critically ill patients with ARF, regardless of whether an AV 1000S or an AV 600S filter is employed. Using a haemodiafiltration dose of 35 mL/kg/h, significant decreases in creatinine and BUN levels were documented during CVVHDF lasting 35 hours. However, repeated clotting was a common problem and almost half of the included patients required continuation of CVVHDF in predilution technique or with other anticoagulation modalities. Significant drops in platelet count and haemoglobin level were a common finding during treatment with CVVHDF. As expected, and in line with the published literature, overall mortality was high and 33.3% of all included patients died during ICU admission and 42.4% during the whole hospital admission, respectively [7, 16, 19–22].

Hypothetically, the use of a filter with a larger membrane surface may turn in being beneficial due to enhanced interleukin-6 removal and reduced resistance, facilitating blood flow through the filtrating membrane and leading to reduced extraction of platelets from the circuit. However, our study did not show any significant differences in filter longevity, bleeding complications, or need for blood transfusions [23–26]. Furthermore, there was visible clot formation in almost all haemodiafiltration circuit components, including the venous bubble-trap chamber and the arterial and the venous lines, suggesting that the overall filtrating system surface, not the filter only, can induce repetitive clotting [26]. We can therefore conclude that the size of the effective filtrating surface area and the blood filling volume are not the only determinants of repetitive clotting and filter related complications. Despite its efficacy, postdilution CVVHDF can lead to significant haemoconcentration, potentially causing higher clotting frequency if compared with continuous venovenous haemodialysis (CVVHD) in predilution technique. Limited data suggest higher filter longevity in CVVHD compared to the median filter longevity documented in our study [27]. However, there is a lack of literature comparing the two different techniques with regard to frequency of clotting and filter related complications in ICU patient with ARF.

The definition of an optimal anticoagulation strategy providing longer circuit patency by minimizing potential harms for the patient plays a crucial role in the setting of CVVHDF. In our study, anticoagulation was performed with unfractionated heparin using a dosing schema similar to the algorithm recommended by Ostermann et al. [28]. Unfractionated heparin has low costs and can be easily monitored in critically ill patients and its effect can be antagonized with protamine. A review of the literature suggests that low dose anticoagulation with unfractionated heparin targeting systemic aPTT values <45 s has low risk for additional bleeding in patients undergoing CRRT, but laboratory and circuit monitoring should be performed frequently in light of the several individual factors that influence the effectiveness and safety of heparin anticoagulation [29, 30]. Point-of-care monitoring of systemic and postfilter aPTT can be used to rapidly adjust heparin dosing. However, according to our experience, achieving target aPTT in critically ill patients is generally difficult, presumably as consequence of their inflammatory response and hypercoagulable state. Furthermore, aPTT is reported to be poor predictor for bleeding complications [31].

In our study, major bleeding complications were not documented, but high-risk patients were primarily treated with other anticoagulation regimes, for example, citrate anticoagulation, and excluded from the observation. Regional citrate anticoagulation has become a valuable alternative to heparin, especially for patients with higher bleeding risk, and might be associated with longer filter survival and improved patient and renal outcomes [32–35]. However, regional citrate anticoagulation is considerably more expensive than heparin and requires intensive monitoring of citrate accumulation and acid-base imbalances.

Hence, the need of an individualised approach in relation to the choice of anticoagulation regimen and its intensity has to be emphasised, considering the high morbidity of the critically ill patients undergoing CVVHDF.

Among the numerous CVVHDF related complications reported in the literature [9, 15–17, 36], we predominantly documented a significant drop in platelet count and haemoglobin level, the last leading to blood transfusion with associated risk of adverse transfusion related events [37, 38]. Our findings concerning the amount or blood transfusions required during CVVHDF reflected those of previous studies [34]. In addition to bleeding due to spillover of regional extracorporeal circuit, heparinisation or disease associated coagulopathy, filter induced haemolysis, and blood loss due to repeated sudden clotting of extracorporeal circuit may have contributed to the haemoglobin drop seen in our patients. An aPTT > 45 s was documented in a large proportion of our patients before and after CRRT initiation. Under these conditions, it is likely that occult gastrointestinal bleeding may be the source of blood loss. Since none of the included patients manifested significant haemolysis, the repeated clotting of the extracorporeal circuit may have caused the blood loss.

We also observed a clinically relevant drop in platelet count, most likely due to platelet activation and consumption in the extracorporeal circuit [25, 39]. The magnitude of platelet loss observed in our study is close to that described in patients with cardiogenic shock and acute renal failure in need of CRRT [39]. Although a median drop of 55,000/*μ*L is clinically relevant, none of our study patients needed platelet transfusion; this is mainly because baseline platelet counts were normal. Since thrombocytopenia is frequent [25] in ICU patients, CRRT induced platelet loss may contribute to an increased risk of bleeding and need for platelet transfusion, potentially leading to a further increase in overall costs. A patient individualized strategy to prevent repeated extracorporeal circuit clotting and to reduce platelet loss is thus warranted [39].

This study has several limitations, first of all the retrospective design. For this reason, both the ICU health-care personnel and the research staff were not blinded for haemodiafiltration settings, anticoagulation adjustments, or data collection. Additionally, the limited number of included patients could have led to an underestimation of the differences between the two filter groups and to inadequate understanding of the clotting mechanisms. In respect of anticoagulation and its monitoring, we could not appropriately compare the course after six hours because of unavailable or irregularly collected laboratory results. Finally, we defined no control group for comparison of filter performance, for example, different filter manufacturer, citrate anticoagulation, or intermittent haemodialysis.

## 5. Conclusion

In summary, this study confirms that CVVHDF with heparin anticoagulation is very effective but complicated by repeated clotting and relevant consumption in platelets and red blood cells. In this setting, the type of filter did not result in any significant difference. However, the retrospective character of the study, the small number of included patients, and observational bias could have led to underestimation of the differences between the filters. Patients with ARF undergoing CVVHDF remain at high risk for unfavourable outcome. Hence, better understanding of underlying mechanisms of clot formation during CVVDHF is needed to improve CVVHDF safety and reduce filter related complications.

## Figures and Tables

**Table 1:** Baseline patient characteristics and laboratory parameters at CVVHDF start.

	All patients (*n* = 33)	Filter AV 1000S (*n* = 17)	Filter AV 600S (*n* = 16)	*P* value
Age—yr	68 (35–87)	68 (35–87)	67 (35–86)	0.82
Male sex—number (%)	21 (64%)	11 (65%)	10 (63%)	0.90
BMI—kg/m^2^	24.6 (14.6–41.5)	26.7 (18.3–41.5)	20.8 (14.6–37.7)	0.07
SOFA score	12 (8–16)	12 (8–15)	12 (8–16)	0.58
SAPS II score	63 (30–98)	63 (30–98)	60 (37–94)	0.87
Noradrenaline—number (%)	23 (69.7%)	13 (76.5%)	10 (62.5%)	0.38
Mechanical ventilation—number (%)	24 (72.7%)	13 (76.5%)	11 (68.7%)	0.56
Admission diagnoses—number (%)				0.33
Cardiovascular	10 (30.3%)	7 (41.2%)	3 (18.8%)	
Sepsis	9 (27.3%)	5 (29.4%)	4 (25.0%)	
Respiratory	4 (12.1%)	1 (5.9%)	3 (18.8%)	
Others	10 (30.3%)	4 (23.5%)	6 (37.4%)	
Length of ICU stay—days	8 (1–168)	8 (3–44)	8 (1–168)	0.79
Length of hospital stay—days	22 (2–171)	19.5 (3–61)	25.5 (2–171)	0.53
ICU mortality—*n*. (%)	11 (33.3%)	7 (41.2%)	4 (25.0%)	0.33
Hospital mortality—*n* (%)	14 (42.4%)	8 (47.1%)	6 (37.5%)	0.58
Creatinine—umol/L	299 (26–909)	320 (210–649)	262 (26–909)	0.28
BUN—mmol/L	21.9 (2.8–40.1)	20.1 (2.8–40.1)	22.1 (3.8–37.3)	0.68
Potassium—mmol/L	4.8 (3.2–6.7)	5.3 (3.9–6.7)	4.6 (3.2–6.4)	0.03
pH	7.30 (6.86–7.49)	7.30 (7.04–7.44)	7.28 (6.86–7.49)	0.64
Lactate—mmol/L	1.2 (0.5–14.7)	1.5 (0.5–4.3)	1.1 (0.5–14.7)	0.46
Bicarbonate—mmol/L	17.9 (6.1–29.8)	19.5 (11.1–29.8)	16.3 (6.1–29.3)	0.18
Platelet count—1/uL	189,000 (47,000–522,000)	189,000 (95,000–522,000)	191,500 (47,000–422,000)	0.59
Haemoglobin—g/dL	8.9 (6.7–15.3)	9.1 (7.7–10.2)	8.9 (6.7–15.3)	0.82
Renal function before admission to ICU^‡^				0.42
GFR > 60 mL/min	15 (45.5%)	9 (52.9%)	6 (37.5%)	
GFR < 60 mL/min	17 (51.5%)	8 (47.1%)	9 (56.3%)	

Results are given as median (range) or percentages; BMI: body mass index; SOFA: sequential organ failure assessment; BUN: blood urea nitrogen; GFR: glomerular filtration rate.

^‡^Total number of patients = 32; filter AV 1000S = 17; filter AV 600S = 15.

**Table 2:** Filter longevity and CVVHDF setting.

	All patients (*n* = 33)	Filter AV 1000S (*n* = 17)	Filter AV 600S (*n* = 16)	*P* value
Numbers of filters—number	2 (1–4)	2 (1–4)	2 (1–4)	0.94
CVVHDF observation period—hours	35 (7–128)	33 (7–128)	39 (7–97)	0.68
Filter longevity—hours	17 (4–67)	15 (4–67)	21 (4–42)	0.63
Haemodiafiltration rate—mL/kg/h	35 (35–80)	35 (35–50)	35 (35–80)	0.75
Ultrafiltration rate—%	17 (9–24)	17 (12–24)	17 (9–20)	0.90
Blood flow—mL/min	150 (100–240)	150 (100–230)	150 (100–240)	0.50
Heparin dose first 6 hours—U/h	450 (0–1050)^¶^	500 (0–1050)	400 (250–900)	0.17
Heparin dose first 6 hours/body weight—U/h/kg	7.1 (0–9.1)^¶^	7.3 (0–9.1)	6.7 (3.4–8.6)	0.17
Absolute creatinine drop—umol/L	171 (8–830)^‡^	221 (97–562)	157 (8–830)	0.48
Relative creatinine drop—%	64 (7–91)	67 (29–87)	69 (7–91)	0.98
Absolute BUN drop—mmol/L	9.5 (0–30.8)	7.3 (0–30.5)	11 (0–30–8)	0.64
Relative urea drop—%	53 (0–85)	53 (0–77)	54 (0–85)	0.54

Results are given as median (range) or percentages.

^¶^Total number of patients = 28; filter AV 1000S = 15; filter AV 600S = 13.

^‡^Total number of patients = 27; filter AV 1000S = 13; filter AV 600 = 14.

## List of Abbreviations

aPTT: Activated partial thromboplastin time
ARF: Acute renal failure
BUN: Blood urea nitrogen
CRRT: Continuous renal replacement therapy
CVVHD: Continuous venovenous haemodialysis
CVVHDF: Continuous venovenous haemodiafiltration
HIT: Heparin induced thrombocytopenia
ICU: Intensive care unit
RRT: Renal replacement therapy
SAPS II: Simplified acute physiology score II
SOFA: Sequential organ failure assessment
U: Unit.
